# Up-regulation of Long Non-coding RNA *TUG1* in Hibernating Thirteen-lined Ground Squirrels

**DOI:** 10.1016/j.gpb.2016.03.004

**Published:** 2016-04-27

**Authors:** Jacques J. Frigault, Daneck Lang-Ouellette, Pier Morin

**Affiliations:** Department of Chemistry and Biochemistry, Faculty of Sciences, Université de Moncton, Moncton E1A 3E9, Canada

**Keywords:** Hibernation, Hypometabolism, Cold adaptation, Non-coding RNAs, lncRNAs

## Abstract

Mammalian **hibernation** is associated with multiple physiological, biochemical, and molecular changes that allow animals to endure colder temperatures. We hypothesize that long **non-coding RNAs** (**lncRNAs**), a group of non-coding transcripts with diverse functions, are differentially expressed during **hibernation**. In this study, expression levels of **lncRNAs***H19* and *TUG1* were assessed via qRT-PCR in liver, heart, and skeletal muscle tissues of the hibernating thirteen-lined ground squirrels (*Ictidomys tridecemlineatus*). *TUG1* transcript levels were significantly elevated 1.94-fold in skeletal muscle of hibernating animals when compared with euthermic animals. Furthermore, transcript levels of *HSF2* also increased 2.44-fold in the skeletal muscle in hibernating animals. *HSF2* encodes a transcription factor that can be negatively regulated by *TUG1* levels and that influences heat shock protein expression. Thus, these observations support the differential expression of the *TUG1*–*HSF2* axis during **hibernation**. To our knowledge, this study provides the first evidence for differential expression of **lncRNAs** in torpid ground squirrels, adding **lncRNAs** as another group of transcripts modulated in this mammalian species during **hibernation**.

## Introduction

Many small mammals undergo hibernation when confronted with unfavorable environmental conditions such as cold temperatures. Hibernation is characterized by a marked reduction in metabolism, prolonged periods where body temperatures (Tb) are significantly reduced (Tb ∼ 4 °C), a substantial reduction in heart rate, as well as resistance to skeletal muscle atrophy [1], [2], [3], [4]. Activity of several metabolic pathways are tightly controlled under these conditions notably via reversible protein phosphorylation of key regulatory enzymes [5]. Regulation of ATP-consuming processes such as gene transcription and protein translation is also a common theme observed in mammalian hibernation [6], [7], [8]. Molecular levers that are utilized to impact these two processes include, for example, histone deacetylases (HDACs) [9] and microRNAs (miRNAs) [10]. Nevertheless, the complete characterization of molecular players underlying mammalian hibernation is ongoing.

Long non-coding RNAs (lncRNAs) are non-coding RNAs (ncRNAs) typically longer than 200 nucleotides that affect diverse cellular functions including gene transcription and protein translation. lncRNAs have been notably shown to impact histone modifications [11], modulate transcription factor–promoter interaction [12], and influence mRNA stability [13]. Interestingly, several lncRNAs were shown to contain miRNA binding sites that could promote miRNA sequestration and subsequent inhibition of miRNA-mediated target recognition and expression [14], [15]. Differential expression of lncRNAs have been reported in a variety of conditions and processes relevant to hibernation including fasting and lipid metabolism [16], [17]. However, identification of torpor-associated lncRNAs has not been fully explored.

Since lncRNAs are involved in regulating crucial processes impacted during mammalian hibernation, the current study was conducted to evaluate the expression of two lncRNAs, *H19* and *taurine up-regulated gene 1* (*TUG1*), in liver, heart, and skeletal muscle tissues of the hibernating thirteen-lined ground squirrels (*Ictidomys tridecemlineatus*). *H19* is one of the earliest lncRNAs identified [18], while *TUG1* can influence the expression and activity of transcription factors relevant to hibernation [19], [20]. We report up-regulation of lncRNA *TUG1* levels in the skeletal muscle of hibernating thirteen-lined ground squirrels and discuss the potential significance of this modulation in mammalian hibernation.

## Results

### Amplification of lncRNAs in thirteen-lined ground squirrels

Consensus sequences of *H19* and *TUG1* in mammalian species were generated for primer design. Target lncRNAs were subsequently amplified in liver, heart, and skeletal muscle tissues of ground squirrels via RT-PCR. The products PCR were confirmed by sequencing and the resulting *H19* and *TUG1* sequences were submitted to GenBank (GenBank accession Nos. KT305775 and KT305776). Figure S1 shows the nucleotide sequences of *H19* and *TUG1* aligned with the sequences for the human, mouse and rat lncRNAs. The partial *H19* nucleotide sequence of ground squirrels displayed 75% homology with that of humans over the amplified region, respectively (Figure S1A). Similarly, the partial *TUG1* nucleotide sequence was 86% homologous with that of humans over the amplified fragment. BLAST alignment of the full length human *TUG1* (GenBank accession No. NR_110492.1) and thirteen-lined ground squirrel genome (SpeTri2.0 reference Annotation Release 101) revealed 74% conservation between sequences from humans and thirteen-lined ground squirrels (GenBank accession No. NW_004936523.1, scaffold 00055).

### *H19* and *TUG1* expression in tissues of hibernating ground squirrels

**Figure 1:**
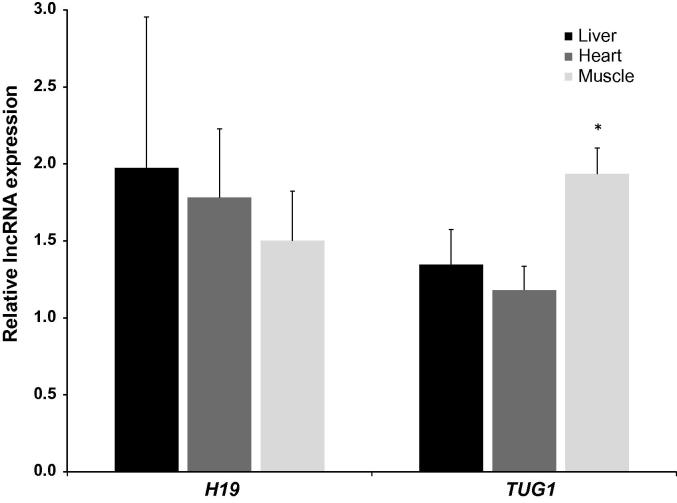
**Relative expression of *H19* and *TUG1* in hibernating ground squirrels** Histogram shows the ratios of normalized lncRNA expression levels against levels of *α-tubulin* measured via qRT-PCR in tissues from hibernating animals compared to euthermic animals. Data are standardized transcript levels (mean ± SEM, *n* = 6 biological replicates) in tissues from hibernating animals relative to those of the same lncRNA in tissues from euthermic animals. Significant difference from euthermic samples is indicated with an asterisk (*t*-test; *P* < 0.005).

*H19* and *TUG1* transcript levels were examined in liver, heart, and skeletal muscle tissues of euthermic and hibernating ground squirrels. Relative levels of both transcripts were quantified in each tissue using qRT-PCR by normalization against that of *α-tubulin* in each sample. Figure 1 shows the ratio of normalized *H19* and *TUG1* transcript levels in the three tissues examined. Compared to euthermic animals, expression levels of *TUG1* in the skeletal muscle of hibernating ground squirrels were 1.94 ± 0.17-fold of that from euthermic animals, which represents a significant increase (*P* < 0.005). On the other hand, although there is a trend of increased expression of *H19* in hibernating animals, the changes are not significant due to the huge variations.

### *HSF2* expression in tissues of hibernating ground squirrels

**Figure 2:**
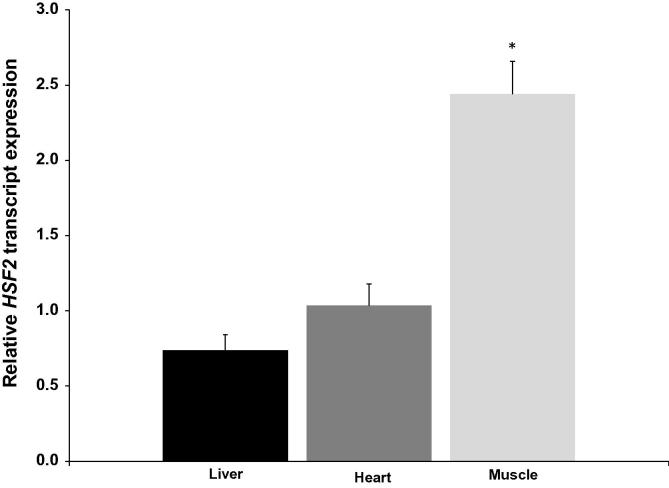
**Relative expression of *HSF2* in hibernating ground squirrels** Histogram shows the ratios of normalized transcript levels of *HSF2* against levels of *α-tubulin* measured via qRT-PCR in tissues from hibernating animals compared to euthermic animals. Data are standardized transcript levels (mean ± SEM, *n* = 6 biological replicates) in tissues from hibernating animals relative to those in tissues from euthermic animals. Significant difference from euthermic samples is indicated with an asterisk (*t*-test; *P* < 0.005).

The transcription factor heat shock factor 2 is encoded by *HSF2*, which is potentially modulated by *TUG1* via miR-144 [21]. We thus measured *HSF2* expression in liver, heart and skeletal muscle tissues of euthermic and hibernating animals using qRT-PCR by normalization against that of *α-tubulin* as above. Figure 2 shows the ratio of normalized *HSF2* transcript levels in all tissues. *HSF2* levels increased by 2.44 ± 0.22-fold in hibernating versus euthermic skeletal muscle tissues (*P* < 0.005). Compared to euthermic animals, expression levels of *HSF2* in the skeletal muscle of hibernating animals were 2.44 ± 0.22-fold of that from euthermic animals, which represents a significant increase (*P* < 0.005), whereas the expression in heart and liver samples were comparable between euthermic and hibernating animals.

### miR-144 expression in skeletal muscle of hibernating ground squirrels

Previous work indicated that *TUG1* can affect *HSF2* expression via miR-144 in glioma cells [21]. Transcript levels of miR-144 were quantified in skeletal muscle tissues of ground squirrel using qRT-PCR. Expression of miR-144 in hibernating animals was 1.31 ± 0.30-fold of that in euthermic animals. However, this change was not statistically significant (*P* > 0.05).

### miR-144 binding site in human and ground squirrel *TUG1* sequences

The miR-144 binding site in human *TUG1* has been reported previously [21]. Interestingly, there is 74% homology between full length human *TUG1* sequence and the whole genome shotgun sequence of the ground squirrels (contig043218; GenBank accession No. AGTP01043218.1). Sequence alignment also revealed a conserved (82.6%) miR-144 binding site in the ground squirrel sequence (Figure S2), suggesting there might exist *TUG1*–miR-144 interaction in the ground squirrels as for humans.

## Discussion

Differential expression of ncRNAs in animal models of cold adaptation has garnered significant interest in the field over recent years. Modulation in expression of ncRNAs, in particular miRNAs, at low temperatures has been reported in small mammalian hibernators [22], [23], cold-hardy insects [24], and freeze-tolerant wood frogs [25]. Unlike miRNAs, lncRNAs have not been explored extensively in models of cold adaptation. Pioneering work in this area has revealed reduced levels of, a natural antisense lncRNA transcript of the gene encoding hypoxia inducible transcription factor-1 alpha (HIF-1α), *aHIF*, in skeletal muscle of torpid little brown bats (*Myotis lucifugus*) [26] and has highlighted the potential translational regulation of HIF-1α during torpor by *aHIF*. Nevertheless, expression data on additional lncRNAs in bats and in other models of cold adaptation is lacking. In this study, we investigated the expression of lncRNAs *H19* and *TUG1* during hibernation in the thirteen-lined ground squirrel and reported differential expression of *H19* in torpid skeletal muscle tissues.

This study reports for the first time *H19* amplification and quantification in a mammalian hibernator. The lncRNA *H19* is a 2.3-kb cytoplasmic transcript that, despite being capped, spliced and polyadenylated, is not translated into a protein [18]. Partial *H19* sequence amplified in this study demonstrated appreciable sequence homology with known *H19* sequences amplified from mammalian models. *H19* can act as a molecular decoy to sequester and regulate the levels of *let-7* [15], a miRNA that regulates several target genes in the insulin–PI3K–mTOR pathway such as *INSR* encoding insulin receptor and *IGF1R* encoding the insulin-like growth factor 1 receptor [27], [28]. Through *let-7* modulation, *H19* levels have also been reported to influence glucose homeostasis in human and rodent models [29]. Interestingly, modulation of the insulin–PI3K–mTOR signaling cascade has been previously reported in torpid ground squirrels and little brown bats [30], [31]. However, our study revealed no significant change in *H19* levels between euthermic and hibernating ground squirrels in liver, heart, and skeletal muscle samples examined. This observation appears to not support *H19* involvement in mammalian hibernation.

**Figure 3:**
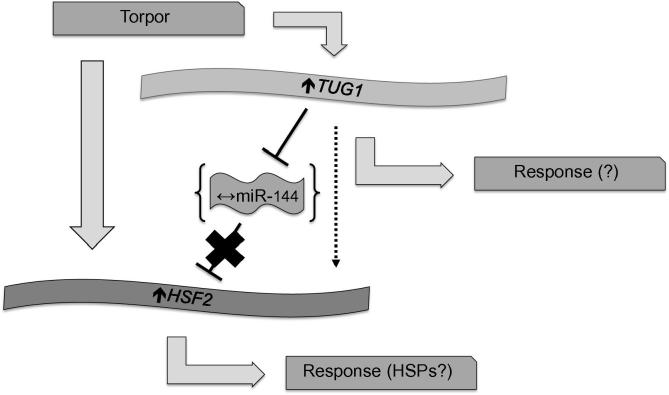
**Proposed working model of the *TUG1*–*HSF2* axis** Torpor leads to up-regulation of *TUG1* and *HSF2* expression in the skeletal muscle of hibernating ground squirrels. miR-144 levels remain unchanged under the same conditions suggesting that *TUG1* impact in torpor is likely via a miR-144-independent mechanism. HSP, heat shock protein.

*TUG1* amplification and quantification were also undertaken for the first time in a hibernating model. Early work on *TUG1* reported substantial expression in adult rodent tissues and demonstrated its likely involvement in retinal development [32]. Recently, there is accumulating evidence supporting *TUG1* implication in various types of cancer including hepatocellular [20], esophageal squamous cell [33], and non-small cell lung [19] carcinomas. Interestingly, *TUG1* was strongly expressed in glioma vascular endothelial cells and modulated expression of *HSF2* by acting as a competitive RNA, or decoy, for miR-144 [21]. Our current work reported *TUG1* up-regulation in hibernating ground squirrel skeletal muscle tissues when compared with euthermic samples. Notably, *HSF2* transcript levels were also increased, pointing toward a potential modulation of a *TUG1*–*HSF2* axis in the skeletal muscle of torpid ground squirrels. HSF2 can transcriptionally regulate expression of different heat shock proteins (HSPs) [34] and there exists interplay between HSF2 and HSF1, a key driver of HSP expression [35]. Interestingly, elevated HSP levels have been reported in various models exposed to cold temperatures including the freeze-tolerant gall fly (*Eurosta solidaginis*) and the larvae of the freeze-tolerant midge (*Belgica antarctica*) [36], [37]. To figure out whether *TUG1* differential expression impacts *HSF2* levels via miR-144, we measured miR-144 transcript levels in skeletal muscle tissues of hibernating ground squirrels. However, no significant change in miR-144 expression was observed, suggesting that *HSF2* up-regulation likely occurs in a miR-144-independent manner (Figure 3).

It is important to point out that the miR-144 binding site observed in human *TUG1* [21] appears to be conserved in ground squirrel *TUG1* (Figure S2). While this observation supports the likely capability of *TUG1* to act as a molecular decoy for miR-144, such interaction might not necessarily lead to the differential expression of miR-144 during torpor in ground squirrels. Regulation of miR-144 levels by *TUG1* in ground squirrel thus warrants further investigation. In general, it will be interesting to build upon these results by investigating the likely molecular effectors that are affected by *TUG1* differential expression and that are modulated downstream of a *TUG1*–miR-144 axis during torpor including miRNAs with predicted *TUG1* binding sites.

Overall, the current work is one of the first studies to report lncRNA differential expression during torpor. Results gathered here thus provide novel insights into the function of ncRNAs, and especially lncRNAs, in mammalian models of hypometabolism. Future work will concentrate on the analysis of additional lncRNAs with potential relevance to cold adaptation as well as on the elucidation of the role of *TUG1* in mammalian hibernation.

## Materials and methods

### Animals

Experimental procedures on thirteen-lined ground squirrels were performed as described before [38]. Squirrels weighing 150–300 g were captured by a trapper (TLS Research, Bloomingdale, IL) and transported to the National Institutes of Health (NIH) facility (Bethesda, MD) of the United States. Animals were kept in shoebox cages maintained at 21 °C and fed *ad libitum* until they accumulated sufficient lipid stores to enter torpor. Experimental procedures conducted on ground squirrels were reviewed and approved by the National Institute of Neurological Disorders and Stroke (NINDS) Animal Care and Use Committee. A sensor chip was injected subcutaneously in anaesthetized animals. Squirrels were moved to a dark cold room maintained at 4 °C–6 °C to induce hibernation. Tb and respiration rate were regularly monitored to identify the stage of torpor-arousal cycle. The animals constituting the hibernating group were sacrificed following a torpor period of at least five days (Tb 5 °C–8 °C), while the control animals had demonstrated stable Tb readings (34 °C–37 °C) for at least three days. All squirrels were sacrificed by decapitation. Tissues were shipped on dry ice to Université de Moncton in New Brunswick and stored at −80 °C until use.

### Total RNA isolation and cDNA synthesis

Total RNA was isolated from 100 mg of liver, heart, and skeletal muscle tissues of ground squirrels using TRIzol reagent (Thermo Fisher Scientific, Ottawa, ON, Canada) as described previously [39]. RNA concentration and purity were evaluated with a NanoVue Plus Spectrophotometer (VWR International, Mississauga, ON, Canada) and RNA samples were kept at −80 °C until use. First strand synthesis was next performed with 1 μg of total RNA using M-MLV reverse transcriptase (Thermo Fisher Scientific) and following manufacturer’s instructions. Serial dilutions of the synthetized cDNA were prepared in water and were used for PCR amplification of the different targets.

### qRT-PCR amplification

Primers for target transcripts in ground squirrels were conceived using a consensus alignment sequence of known corresponding mammalian transcripts. Primer sequences are displayed in Table S1. *H19*, *TUG1*, and *HSF2* were amplified via RT-PCR as described before [39] and PCR products were validated by sequencing. Subsequently, qRT-PCR was performed using iTaq Universal SYBR Green (Bio-Rad, Mississauga, ON, Canada) as described previously [40]. Amplification consisted of an initial step at 95 °C for 5 min, followed by 40 cycles at 95 °C for 15 s, optimal annealing temperature for 30 s and 72 °C for 60 s. *α-tubulin* was used as a housekeeping control. miR-144 transcript levels were quantified by qRT-PCR as described before, using miR-107 transcript levels as housekeeping control [10].

### Quantification and statistical analysis

Quantification cycle (Cq) values were collected using the Bio-Rad CFX Manager software. Levels of target transcripts were normalized against those of *α-tubulin* amplified from the same cDNA sample. Transcript quantification was performed using the 2^−ΔΔCt^ method [41]. Ratios of normalized transcript levels in hibernating samples to normalized transcript expression in euthermic samples were obtained. Significant differences (*P* < 0.005) between the two groups were evaluated with Student’s *t*-test.

## Authors’ contributions

JF performed the experiments and wrote parts of the manuscript. DLO designed the PCR primers. PJM conceived the study, supervised the project, and wrote most of the manuscript. All authors read and approved the final manuscript.

## Competing interests

The authors declare no conflict of interests.

## Supplementary material

Supplementary Figure S1 Alignment of partial *H19* and *TUG1* nucleotide sequences across different species. *H19* (**A**) and *TUG1* (**B**) sequences amplified from ground squirrels were aligned with corresponding sequences of the human, mouse and rat. GenBank accession numbers are NR_002196.2 (human), NR_130973.1 (mouse), and NR_027324.1 (rat) for *H19*, and NR_002323.2 (human), NR_002321.2 (mouse), and NR_130147.1 (rat) for *TUG1*, respectively. Spacer dots indicate missing nucleotides in one or more sequence.

Supplementary Figure S2 miR-144 binding site in *TUG1* sequences from human and ground squirrel. miR-144 potential binding site is found in human *TUG1* (NR_110492.1, nucleotides 4725−4747) and ground squirrel genome shotgun sequence (AGTP01043218.1 nucleotides 7044−7190). miR-144 binding site is underlined in black and exhibits 82.6% conservation (19 out of 23 nucleotides). Spacer dots indicate missing nucleotides in one sequence.

Supplementary Table S1 Information of the primers used for qRT-PCR in this study.
