# Supplementary material for: Up-regulation of Long Non-coding RNA TUG1 in Hibernating Thirteen-lined Ground Squirrels
Source: Genomics Proteomics Bioinformatics. 2016 Apr 27;14(2):113–8. doi: 10.1016/j.gpb.2016.03.004 (PMC4880950; doi:10.1016/j.gpb.2016.03.004)
Supplement: Supplementary Figure S1 — Alignment of partial H19 and TUG1 nucleotide sequences across different species. H19 (A) and TUG1 (B) sequences amplified from ground squirrels were aligned with corresponding sequences of the human, mouse and rat. GenBank accession numbers are NR_002196.2 (human), NR_130973.1 (mouse), and NR_027324.1 (rat) for H19, and NR_002323.2 (human), NR_002321.2 (mouse), and NR_130147.1 (rat) for TUG1, respectively. Spacer dots indicate missing nucleotides in one or more sequence. [file mmc1.pptx]

## Slide 1
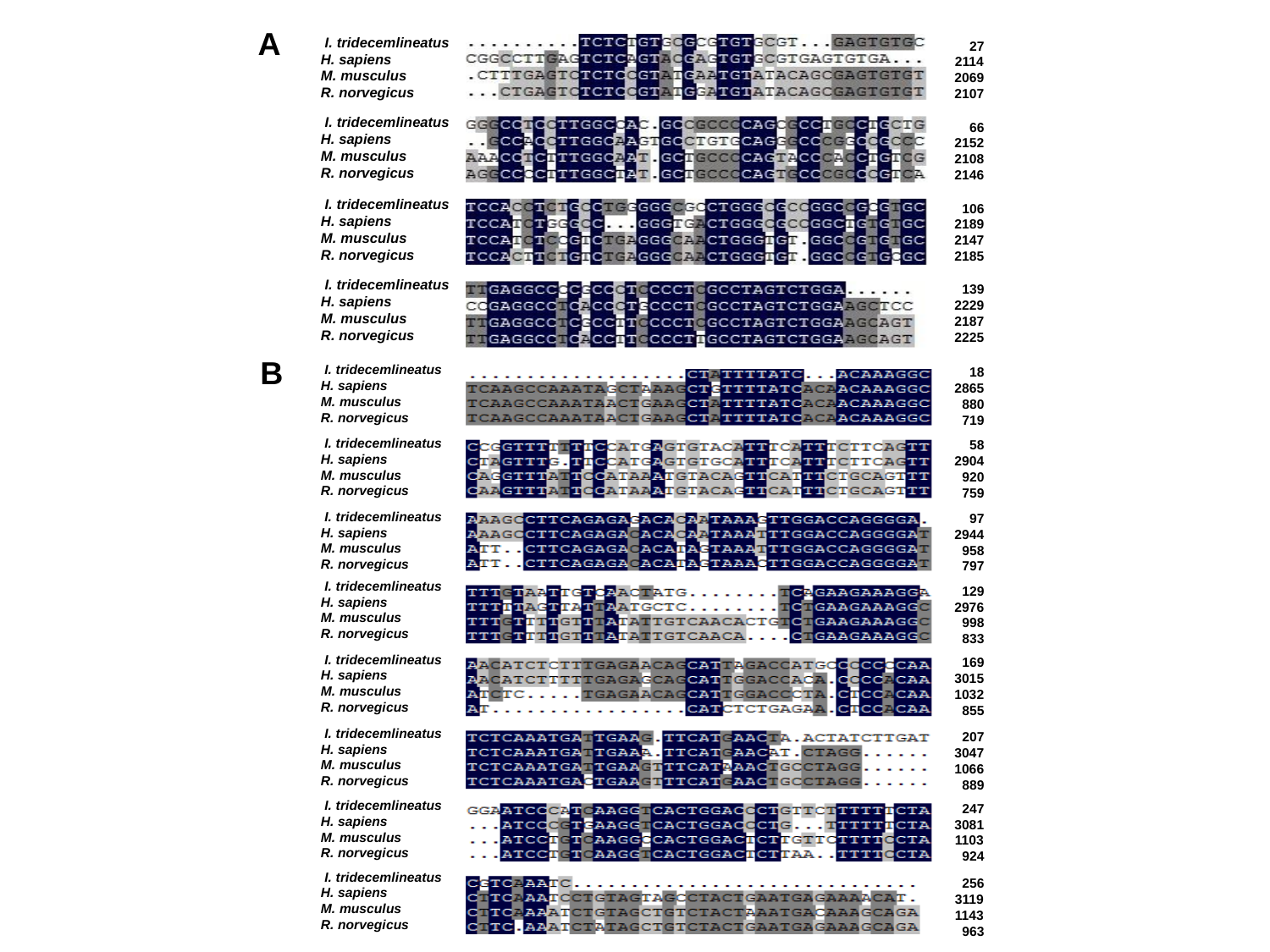

A
 I. tridecemlineatus
H. sapiens
M. musculus
R. norvegicus
 I. tridecemlineatus
H. sapiens
M. musculus
R. norvegicus
 66
2152
2108
2146
 106
2189
2147
2185
 I. tridecemlineatus
H. sapiens
M. musculus
R. norvegicus
 I. tridecemlineatus
H. sapiens
M. musculus
R. norvegicus
 139
2229
2187
2225
 66
2152
2108
2146
 27
2114
2069
2107
 66
2152
2108
2146
 106
2189
2147
2185
 139
2229
2187
2225
B
 I. tridecemlineatus
H. sapiens
M. musculus
R. norvegicus
 18
2865
 880
 719
 I. tridecemlineatus
H. sapiens
M. musculus
R. norvegicus
 58
2904
 920
 759
 I. tridecemlineatus
H. sapiens
M. musculus
R. norvegicus
 97
2944
 958
 797
 I. tridecemlineatus
H. sapiens
M. musculus
R. norvegicus
 129
2976
 998
 833
 I. tridecemlineatus
H. sapiens
M. musculus
R. norvegicus
 169
3015
1032
 855
 I. tridecemlineatus
H. sapiens
M. musculus
R. norvegicus
 207
3047
1066
 889
 I. tridecemlineatus
H. sapiens
M. musculus
R. norvegicus
 247
3081
1103
 924
 I. tridecemlineatus
H. sapiens
M. musculus
R. norvegicus
 256
3119
1143
 963
