# Supplementary material for: Up-regulation of Long Non-coding RNA TUG1 in Hibernating Thirteen-lined Ground Squirrels
Source: Genomics Proteomics Bioinformatics. 2016 Apr 27;14(2):113–8. doi: 10.1016/j.gpb.2016.03.004 (PMC4880950; doi:10.1016/j.gpb.2016.03.004)
Supplement: Supplementary Figure S2 — miR-144 binding site in TUG1 sequences from human and ground squirrel. miR-144 potential binding site is found in human TUG1 (NR_110492.1, nucleotides 4725−4747) and ground squirrel genome shotgun sequence (AGTP01043218.1 nucleotides 7044−7190). miR-144 binding site is underlined in black and exhibits 82.6% conservation (19 out of 23 nucleotides). Spacer dots indicate missing nucleotides in one sequence. [file mmc2.pptx]

## Slide 1
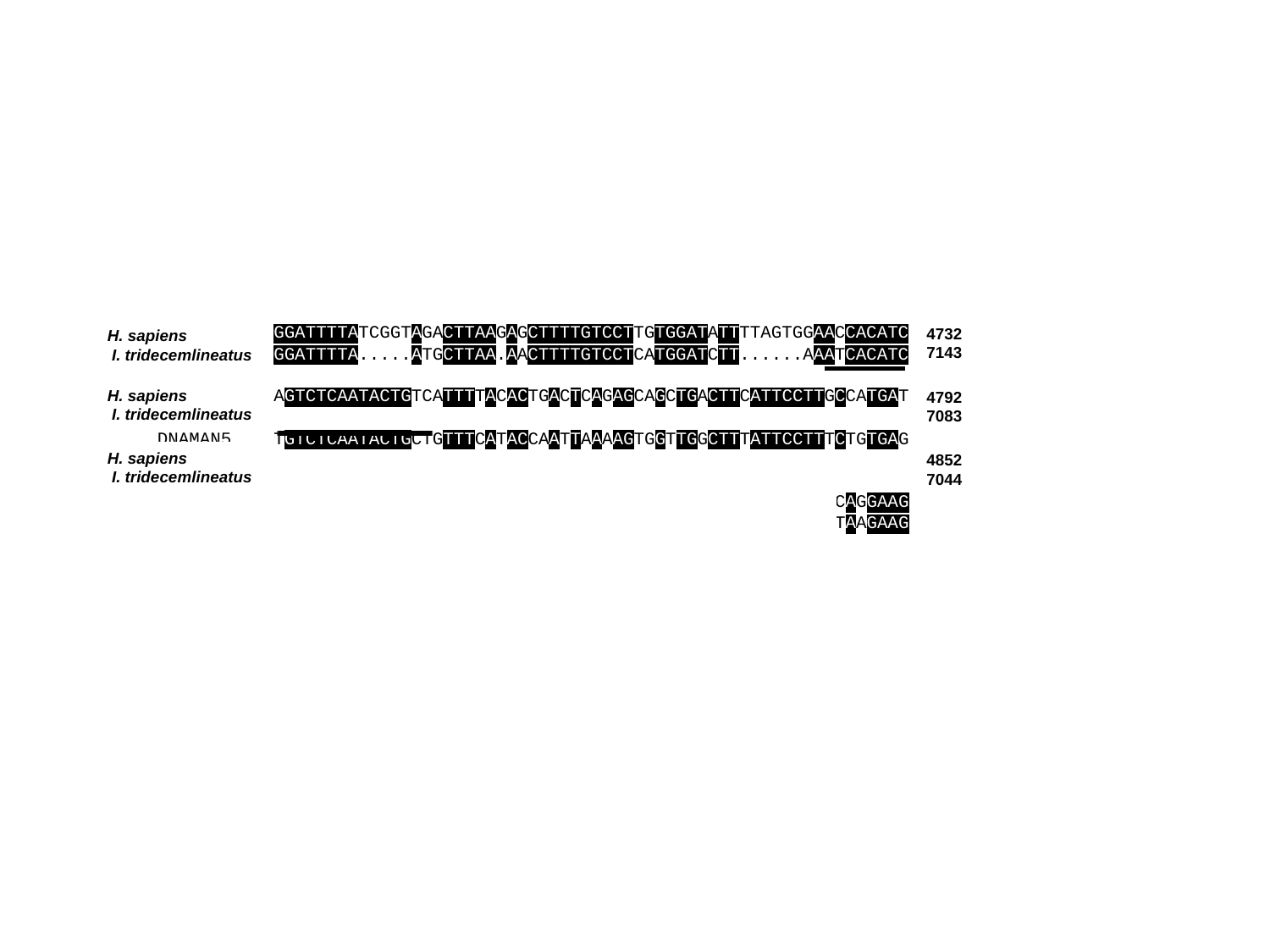

DNAMAN1 GGATTTTATCGGTAGACTTAAGAGCTTTTGTCCTTGTGGATATTTTAGTGGAACCACATC
DNAMAN5 GGATTTTA.....ATGCTTAA.AACTTTTGTCCTCATGGATCTT......AAATCACATC
DNAMAN1 AGTCTCAATACTGTCATTTTACACTGACTCAGAGCAGCTGACTTCATTCCTTGCCATGAT
DNAMAN5 TGTCTCAATACTGCTGTTTCATACCAATTAAAAGTGGTTGGCTTTATTCCTTTCTGTGAG
DNAMAN1 ATATATTTAAGGCAGGCATTGTAACAGACATAAAGACAACTTATCTGTTTCAGCAGGAAG
DNAMAN5 AT...............ACTGT..TAGTCATTAGGACAATTTA....TTTCTGTAAGAAG
DNAMAN1 TATCGGTAGACTTAAGAGCTTTTGTCCTTGTGGATATTTT 4718
DNAMAN5 ........................................ 4302
DNAMAN1 AGTGGAACCACATCAGTCTCAATACTGTCATTTTACACTG 4758
DNAMAN5 .....................................CTG 4305
DNAMAN1 ACTCAGAGCAGCTGACTTCATTCCTTGCCATGATATATAT 4798
DNAMAN5 TCTCAATACTGCTGT.TTCATACCAATTAAAAGTGGTTGG 4344
DNAMAN1 TTAAGGCAGGCATTGTAACAGACATAAAGACAACTTATCT 4838
DNAMAN5 CTTTATTCCTTTCTGTGAGATACTGTTAGTCATTAGGACA 4384
DNAMAN1 GTTTCAGCAGGAAGGATTCAGTTTATGAACTCTCAGACCA 4878
DNAMAN5 ATTTATTTCTGTAAGAAGGATTCAGTTCTTGAATTC.CCA 4423
DNAMAN1 GATCATGTTGAACAAGGAGACTTTGATGTGTGTCATGAGA 4918
DNAMAN5 GATCATATCAAATGAAGAGACTTCGATTTGTGTCCTGAGA 4463
 4732
7143
H. sapiens
 I. tridecemlineatus
H. sapiens
 I. tridecemlineatus
 4792
7083
H. sapiens
 I. tridecemlineatus
 4852
7044
