# Supplementary material for: Up-regulation of Long Non-coding RNA TUG1 in Hibernating Thirteen-lined Ground Squirrels
Source: Genomics Proteomics Bioinformatics. 2016 Apr 27;14(2):113–8. doi: 10.1016/j.gpb.2016.03.004 (PMC4880950; doi:10.1016/j.gpb.2016.03.004)
Supplement: Supplementary Table S1 — Information of the primers used for qRT-PCR in this study. [file mmc3.docx]

**Table S1 Information of the primers used for qRT-PCR in this study**

| **Primer** | | **Sequence** | **Efficiency** | **Optimal annealing temperature** |
| --- | --- | --- | --- | --- |
| *H19* | Forward | 5’- AATTTGCACTAAGTCGATTGC -3’ | 92.1% | 54.7 °C |
|  | Reverse | 5’- GCTTCCAGACTAGGCGAGGG -3’ |  |  |
| *TUG1* | Forward | 5’- CAGATTCAGCACAGCCCTTT -3’ | 95.7% | 57.0 °C |
|  | Reverse | 5’- ATCCCAATGGGTCAGAATAT -3’ |  |  |
| *HSF2* | Forward | 5’- CATCATAAAGTTCCACACAG -3’ | 87.6% | 54.3 °C |
|  | Reverse | 5’- GTTTCTGGAATAACTGG -3’ |  |  |
| *α-tubulin* | Forward | 5’- GCCTTTGTGCACTGGTACG -3’ | 99.6% | 58.0 °C |
|  | Reverse | 5’- TTAGTATTCCTCTCCTTCTTCCTC-3’ |  |  |
| miR-144 | Forward | 5’- ACACTCCAGCTGGGTACAGTATAGATGATG - 3’ | 84.1% | 62.4 °C |
|  | Stem loop | 5’-CTCACAGTACGTTGGTATCCTTGTGATGTTCGAT  GCCATATTGTACTGTGAGAGTACATC - 3’ |  |  |
| miR-107 | Forward | 5’- ACACTCCAGCTGGGAGCAGCATTGTACAGG - 3’ | 97.0% | 60.0 °C |
|  | Stem loop | 5’-CTCACAGTACGTTGGTATCCTTGTGATGTTCGAT  GCCATATTGTACTGTGAGTGATAGCC - 3’ |  |  |
